# Newly identified helper bacteria stimulate ectomycorrhizal formation in *Populus*

**DOI:** 10.3389/fpls.2014.00579

**Published:** 2014-10-24

**Authors:** Jessy L. Labbé, David J. Weston, Nora Dunkirk, Dale A. Pelletier, Gerald A. Tuskan

**Affiliations:** Biosciences Division, Oak Ridge National LaboratoryOak Ridge, TN, USA

**Keywords:** mycorrhiza helper bacteria, mycorrhizal fungi, *Laccaria bicolor*, mutualistic interactions, fungal-bacterial-*Populus* interactions, *Populus* rhizosphere, soil microbiology

## Abstract

Mycorrhiza helper bacteria (MHB) are known to increase host root colonization by mycorrhizal fungi but the molecular mechanisms and potential tripartite interactions are poorly understood. Through an effort to study *Populus* microbiome, we isolated 21 *Pseudomonas* strains from native *Populus deltoides* roots. These bacterial isolates were characterized and screened for MHB effectiveness on the *Populus-Laccaria* system. Two additional *Pseudomonas* strains (i.e., Pf-5 and BBc6R8) from existing collections were included for comparative purposes. We analyzed the effect of co-cultivation of these 23 individual *Pseudomonas* strains on *Laccaria bicolor* “S238N” growth rate, mycelial architecture and transcriptional changes. Nineteen of the 23 *Pseudomonas* strains tested had positive effects on *L. bicolor* S238N growth, as well as on mycelial architecture, with strains GM41 and GM18 having the most significant effect. Four of seven *L. bicolor* reporter genes, *Tra1, Tectonin2, Gcn5*, and *Cipc1*, thought to be regulated during the interaction with MHB strain BBc6R8, were induced or repressed, while interacting with *Pseudomonas* strains GM17, GM33, GM41, GM48, Pf-5, and BBc6R8. Strain GM41 promoted the highest roots colonization across three *Populus* species but most notably in *P. deltoides*, which is otherwise poorly colonized by *L. bicolor*. Here we report novel MHB strains isolated from native *Populus* that improve *L. bicolor* root colonization on *Populus*. This tripartite relationship could be exploited for *Populus* species/genotypes nursery production as a means of improving establishment and survival in marginal lands.

## Introduction

The majority of terrestrial plants are known to form specialized mycorrhizal structures with symbiotic fungi (Read et al., 2000). This mycorrhizal symbiosis enhances plant growth and has been actively studied for its potential use in agriculture and forestry applications. Understanding the ecology and physiology of the association and how to control its establishment and stability has become a key focus in basic and applied plant sciences. In addition, several researchers have noted that the establishment and functioning of mycorrhizal symbioses can be positively influenced by certain bacterial strains that have been categorized as Mycorrhiza Helper Bacteria (MHB) (Garbaye, 1994; Frey-Klett et al., 2007).

Known MHB strains include a variety of Gram-negative (Barea et al., 1998; Founoune et al., 2002b; Frey-klett et al., 2005) and Gram-positive (Budi et al., 1999; Poole et al., 2001; Schrey et al., 2005) bacterial species. Because of their potential to increase plant nutrition and health, the use of MHB in low-input agriculture and forestry has been addressed in several investigations (Duponnois et al., 1993; Barea et al., 1998; Becker et al., 1999; Schrey et al., 2005). The definition of the MHB concept is evolving, encompassing either the type of mycorrhizal symbiosis or the taxonomy of the MHB strains. To date, many bacterial strains have been reported to promote either endo- or ectomycorrhizal symbioses (Garbaye, 1994; Barea et al., 2002; Johannsson et al., 2004; Artursson et al., 2006; Duponnois, 2006; Pivato et al., 2009) and various plant models have been used to study the MHB effect (Garbaye, 1994; Schrey et al., 2005; Duponnois and Kisa, 2006; Pivato et al., 2009). There are, however, no reports on the characterization of MHB strains isolated from a single host species and then subsequently re-inoculated on that host to determine if there is a beneficial response. In this study we used *Populus* spp. (poplar, cottonwood, aspen) to test these relationships.

*Populus* is cultivated worldwide for pulp and paper, veneer, packing material, engineered wood products (e.g., oriented strand board), lumber, and has recently emerged as the preeminent fast-growing woody crop for bioenergy research (Tuskan and Walsh, 2001; Jansson and Douglas, 2007; Sannigrahi and Ragauskas, 2010). *Populus* can be grown on economically marginal agricultural land thereby minimizing the competition between food and fuel production (Bradshaw et al., 2000; Tuskan et al., 2006; Sannigrahi and Ragauskas, 2010). Moreover, *Populus* is known to associate with a wide variety of root symbiotic microbes. *Populus* is also one of the few plants known to be colonized by both endo- and ectomycorrhizal fungi, making it a unique model system for the study of interactions between plants and microorganisms (Martin et al., 2004). To date, the study of the role of bacterial associates of *Populus* has been limited to plant-growth-promoting studies or phytoremediation research (Moore et al., 2006; Taghavi et al., 2010; Weston et al., 2012), with little attention given to their potential role as MHB facilitating *Populus*-fungal interactions.

As part of a recent effort characterizing microbial rhizosphere communities of native *Populus deltoides* (Gottel et al., 2011), a collection of *Populus*–associated bacterial isolates comprising seven classes and 89 genera, as determined by 16S rDNA sequence analysis, has been established (Brown et al., 2012). A subset of 43 isolates has been genome sequenced, of which only 21 are *Pseudomonas* related strains, which were selected here for comparison with the reference helper *Pseudomonas* BBc6R8 (Deveau et al., 2007). Thus, we investigated the effect of potential MHB bacterial isolates from native *P. deltoides* rhizosphere on the ectomycorrhizal *Populus-Laccaria bicolor* interaction. The objectives of this work were to: (1) determine whether the *P. deltoides*-derived bacterial isolates have any effect on *L. bicolor* mycelial architecture and growth, (2) assess the expression level of seven target genes in *L. bicolor* shown to be regulated by the MHB strain BBc6R8 (Deveau et al., 2007), (3) determine whether the diverse fungus-bacteria combinations have any effect on *Populus* root architecture and (4) characterize ectomycorrhizal formation across selected combinations of MHB strains.

## Materials and methods

### Microorganisms, strains, and culture conditions

The symbiotic fungus *Laccaria bicolor* (Maire) P.D. Orton is a member of the Hydnangiaceae (Basidiomycota, Agaricomycotina, Agaricomycetes, Agaricomycetidae, Agaricales), a large family of ectomycorrhizal and saprotrophic basidiomycetes. A subculture of this strain, S238-O, was transferred to the INRA-Nancy in 1980 and renamed S238N (Di Battista et al., 1996). The genomic sequence of the monokaryotic mycelia S238N-H82 was subsequently published (Martin et al., 2008). The dikaryotic mycelium of *L. bicolor* S238N used in this study was grown and maintained in Petri dishes containing Pachlewski agar medium P5 (Di Battista et al., 1996) and incubated at 25°C for 3 week.

The *Pseudomonas fluorescens* strain BBc6R8 was isolated from *L. bicolor* S238N associated with Douglas-fir (*Pseudotsuga menziesii*) roots (Duponnois and Garbaye, 1991). This strain has been categorized as MHB as it increases mycorrhization and/or growth of the fungus (Duponnois and Garbaye, 1991; Frey-Klett et al., 1997; Deveau et al., 2007); it was used as a positive control in our experiments. The specific strain, BBc6R8, used in this study is a spontaneous rifampicin resistant mutant, but phenotypically conforms to the parental strain BBc6. The *Pseudomonas protegens* Pf-5 (previously *P. fluorescens* Pf-5) ATCC BAA-477 (Howell and Stipanovic, 1978; Paulsen et al., 2005) was purchased from American Type Culture Collection (Manassas, VA), is a well-studied biocontrol strain (Loper et al., 2007) and was included as a negative control.

*Pseudomonas* sp. strains GM16, GM17, GM18, GM21, GM24, GM25, GM30, GM33, GM41, GM48, GM49, GM50, GM55, GM60, GM67, GM74, GM78, GM79, GM80, GM84, and GM102, were isolated from *P. deltoides* rhizosphere or surface sterilized roots of mature native trees collected in central Tennessee (Gottel et al., 2011). All the bacterial strains were maintained at −80°C in Luria-Bertani (LB) medium (Sambrook et al., 1989) with 20% glycerol.

### Bacterial-fungal pairwise assays and morphological measurements

From maintenance cultures, each of the bacterial strains were subcultured on 10% TSA [i.e., 15 g l^−1^ of agar, 3 g l^−1^ tryptic soy broth (Difco, Detroit, MI)] plates at 25°C for 65 h to prepare the bacterial inoculum for the *in vitro* bioassay. Then, three to four colonies were picked and suspended in 2 ml of sterile deionized water before re-distribution on to 10% TSA medium. After 48 h of growth at 25°C, the bacteria were harvested and centrifuged at 3300 g for 10 min. The pellet was washed once and then diluted in deionized water in order to obtain a suspension with A600_nm_ 0.7 (ca. 10^9^ cfu ml^−1^).

*In vitro* pairwise assays were performed as reported by Deveau et al. (2007), on 150-mm Petri dishes containing 25 ml of modified Pachlewski medium (P5, i.e., P20Th^−^; 0.5 g tartrate, 1 g KH_2_PO_4_, 0.5 g MgSO_4_, 1 g glucose, 1 ml 1/10 diluted Kanieltra micronutrient solution and 20 g l^−1^ agar, pH 5.5). A 10-mm plug of *L. bicolor* S238N mycelium was cut out from the edge of a colony grown on P5 medium and transferred into the center of a P20Th^−^ plate. Four 10-μl droplets of sterile deionized water (control) or bacterial suspension (treatment) were distributed diametrically opposite at 1.75 cm from the center of the fungal plug. Plates were sealed with electric tape and incubated at 10°C in the dark. The diameter of the fungal colony was measured every fourth day from day 12 to 32 after the addition of water or bacterial suspensions, with seven replicates per treatment. Architecture of the fungal mycelium was analyzed at three key stages: before contact between the mycelium and the bacteria (14 d), at contact time (ca. 16 d) and after an extended contact period (21 d). Three biological replicates (independent experiments) per treatment were performed. For each replicate, two photographs were taken using a Zeiss MicroImaging steREO Discovery V.8 (40x magnification) equipped with a Color-View System AxioCam ICc1 camera (Zeiss, Thornwood, NY). The number of apices per microscopic field (3.5 mm^2^), branching angles, branching densities (number of ramifications divided by the number of apices) and curvature of hyphae were measured on each photograph using the AxioVision 4.8 imaging processing software (Zeiss, Thornwood, NY). The effect of the bacterial treatment on the growth and architecture of the fungal mycelium was determined using analysis of variance (ANOVA) at a *p*-value of 0.05. The Least Significant Difference (LSD) test was used for pairwise comparison after ANOVA. The R statistical package 2.12.1 was used for all statistical analyses (R Foundation for Statistical Computing, Vienna, Austria).

### Gene expression analysis in *L. bicolor*

To verify BBc6R8 effect on *L. bicolor* gene expression (Deveau et al., 2007), and to compare responses among bacterial strains, we analyzed the expression of seven target/reporter genes from *L. bicolor* S238N mycelium in pairwise assays. Mycelium was collected in triplicate before contact between the fungus and bacteria (14 d) and after an extended contact period (21 d). RNA was extracted using the Spectrum Plant Total RNA Kit (Sigma-Aldrich, St. Louis, MO) following the manufacturer's recommendations. The quality of the RNA was checked by RNAse-free 1% agarose electrophoresis. The cDNAs were synthesized using the SuperScript III First-Strand Synthesis System kit (Invitrogen-Live Technologies, Grand Island, NY). Real-time PCR analyses were performed using primers for seven up- and/or down-regulated genes (i.e., *Cipc1, Tectonin2, Tra1, Gcn5, Ada3, Fox2*, and *Spt3*) and two non-regulated genes (i.e., Lb17E10 and trehalose phosphorylase) as controls. Data normalization was as described by Deveau et al. (2007). Real-Time PCRs were carried out on the StepOne RT-PCR system using the SYBR Green DNA detection dye (Applied Biosystems, Foster City, CA). A negative control reaction that did not contain DNA template (“water blanks”) was run for each primer pair to demonstrate absence of PCR products in amplifications of cDNA from fungal tissues. For data analysis, the geometric mean of the three biological replicates for each condition was calculated. The PCR efficiency was checked and fold differences were calculated using the ΔΔCt method (Livak and Schmittgen, 2001).

### Plant material and co-culture experiments

*In vitro* experiments were performed with micropropagated *Populus* clones “717-1B4” (female, *P*. *tremula* × *alba*), “93-968” (female, *P. trichocarpa*), and “D-124” (male, *P. deltoides*). Fresh, actively growing shoots from plants grown in a greenhouse were used as propagation materials and were established in 100 ml of MS medium with micro and macronutrients, vitamins and glycine (Murashige and Skoog, Sigma-Aldrich, St. Louis, MO) supplemented with 1% (w/v) sucrose, 0.5 g MES hydrate (Sigma-Aldrich) and solidified with 0.8% (w/v) agar and pH adjusted to 5.7. Explants were established in culture after surface-sterilization [i.e., 5 min in 1% (v/v) Tween-20 solution, 1 min in 70% (v/v) ethanol, 15 min in 10% (v/v) bleach solution and then triple rinsed for 5 min in sterile water]. The plants were maintained in Magenta boxes (GA-7) and were cultivated in a tissue culture growth room at 25°C with a 24-h photoperiod under fluorescence tubes (cool white, 95 W, F96T12/CW/HO/SS, Sylvania) at an intensity of 70 μmol m^−2^ s^−1^. Plants were multiplied every 6–8 weeks to maintain vigorous stock plants (Kang et al., 2009). Plant materials were simultaneously prepared for *in vitro* co-culture experiments. In order to synchronize rhizogenesis, stem cuttings from the *in vitro* stock plants were pre-cultured on MS medium containing 2 mg l^−1^ indole butyric acid for 1 week. Rooted cuttings were then transferred to vertically arranged 12 × 12 cm Petri dishes half covered with a 6 × 6 cm cellophane membrane and containing sugar-minus Pachlewski medium P20Th- (Deveau et al., 2007). These rooted cutting were inoculated or not (control) according to the following treatments—bacteria alone (BBc6R8, GM17, GM18, GM30 or GM41), fungus alone (*L. bicolor* S238N) or pairwise combinations of bacteria and fungus. Bacterial and fungal inocula were synchronously prepared for the assays. Three 10-μl droplets of sterile deionized water (control) or bacterial suspension or three 10-mm-fungal plugs were horizontally distributed at 1.75 cm from the root system. In the bacterial-fungal treatment, bacterial droplets were vertically distributed at 0.6 cm from the fungal plugs that were spaced of 0.6 cm and vertically distributed at 1.75 cm from the root system. Plates were sealed with tape on the upper and lower sides and with a gas permeable cover on both lateral sides. Sterile dental cotton was placed at the bottom of the plate for attenuating moisture. Plant co-cultures were arranged vertically and the lower part of the dish was covered with a small black plastic bag to prevent light from reaching microbes and roots. The co-cultures were kept in the same environmental conditions as the *in vitro* plant stock above. Quantification of the number of secondary roots and their length at 14 and 31 days post-inoculation (DPI) were performed using WinRHIZO and Photoshop software. The effect of the treatment on the number and length of the secondary roots was determined using ANOVA at a *p*-value of 0.05.

Greenhouse experiments were performed with the same *Populus* clones listed above. Surface-sterilized-internode cuttings of each clone from plants grown in a greenhouse were rooted and individually simultaneously inoculated in 164-ml leach tubes containing a mixture of fungal inoculum (1:9, v:v) and autoclaved peat-vermiculite (1:1, v:v). Solid inoculum was prepared in 1-l glass jars containing 800 ml autoclaved peat-vermiculite mixture (1:1, v:v) moistened with 650 ml liquid Pachlewski medium, inoculated with plugs from an agar culture and incubated for 6 weeks at 25°C (Duponnois and Garbaye, 1991). Bacterial inocula for BBc6R8, GM17, GM18, GM30, and GM41 were prepared as described by Frey-Klett et al. (1997). The absorbance of each bacterial suspension at 600 nm was adjusted to 1 in 0.1 mol l^−1^ MgSO_4_ buffer; then the suspensions were diluted 10 times. Five ml of each bacterial suspension (10^7^ CFU ml^−1^) were distributed on the agar surface immediately after planting; the controls without bacteria received only 5 ml of 0.1 mol l^−1^ MgSO_4_ buffer. One cutting was grown per container. The containers were arranged in trays holding 12 containers (4 replicates per treatment). One tray was inoculated for each bacterial isolate. The control treatments—*Populus* clone-*L. bicolor* and *Populus* clone alone—were contained in three trays (one *Populus* clone per tray). In order to control environmental heterogeneity of the greenhouse, the trays were placed at random on two tables and were re-distributed weekly according to a circular permutation. Inoculated and control cuttings were grown for 12 weeks in the greenhouse at 20–25°C, photoperiod 12 h during fall. Cuttings were watered twice daily with sterile water and received a weekly nutrient application as described in Frey-Klett et al. (1997). Twelve weeks after inoculation, the proportion of short roots forming mycorrhizae was determined by randomly examining 100 short roots per plantlet. Each root system was rinsed with sterile water, cut in 1-cm pieces and analyzed under a stereomicroscope. *L. bicolor* ectomycorrhizae were identified morphologically. The rinse water for each plant was recovered and 1 ml used to re-inoculate five LB plates (Sambrook et al., 1989) at 25°C for 48 h in order to check the presence of the bacterial inoculum.

An individual bacterial colony was used to inoculate 40 ml liquid LB medium (Sambrook et al., 1989) at 25°C for 24 h. Then, bacterial DNA was isolated according to the QIAamp DNA Mini Kit protocol (QIAGEN, Valencia, CA). The 16S rDNAs were amplified by PCR with the primers fD1 and rD1 (Weisburg et al., 1991) and sequenced on an ABI 3730XL sequencer following the Applied Biosystems BigDye v.3.0 sequencing protocol. The 16S rDNA sequences were analyzed using Sequencher 5.0 software (Gene Code Corporation, Ann Arbor, MI). The presence of the bacterial inocula at 12 weeks was confirmed by complete match of the 16S rDNA sequences performed at the beginning and the end of the experiment. The percentage of mycorrhizal colonization was transformed using an arcsine √X/100 function prior to ANOVA. The following mixed linear model was applied on an individual cutting basis to detect significant differences in mycorrhizal colonization among the clones and the bacterial treatment:

$${\text{Y}}_{\text{ijkl}}=\mu +{\text{B}}_{\text{i}}+{\text{G}}_{\text{j}}+{\text{T}}_{\text{l}}+\varepsilon_{\text{ijkl}}$$

where, μ is the overall mean, B is the block effect (fixed), G is the genotype effect (random), T is the bacterial treatment (random) and ε is the random pooled residual error.

## Results

### *L. bicolor* mycelial growth and architecture

**Figure 1:**
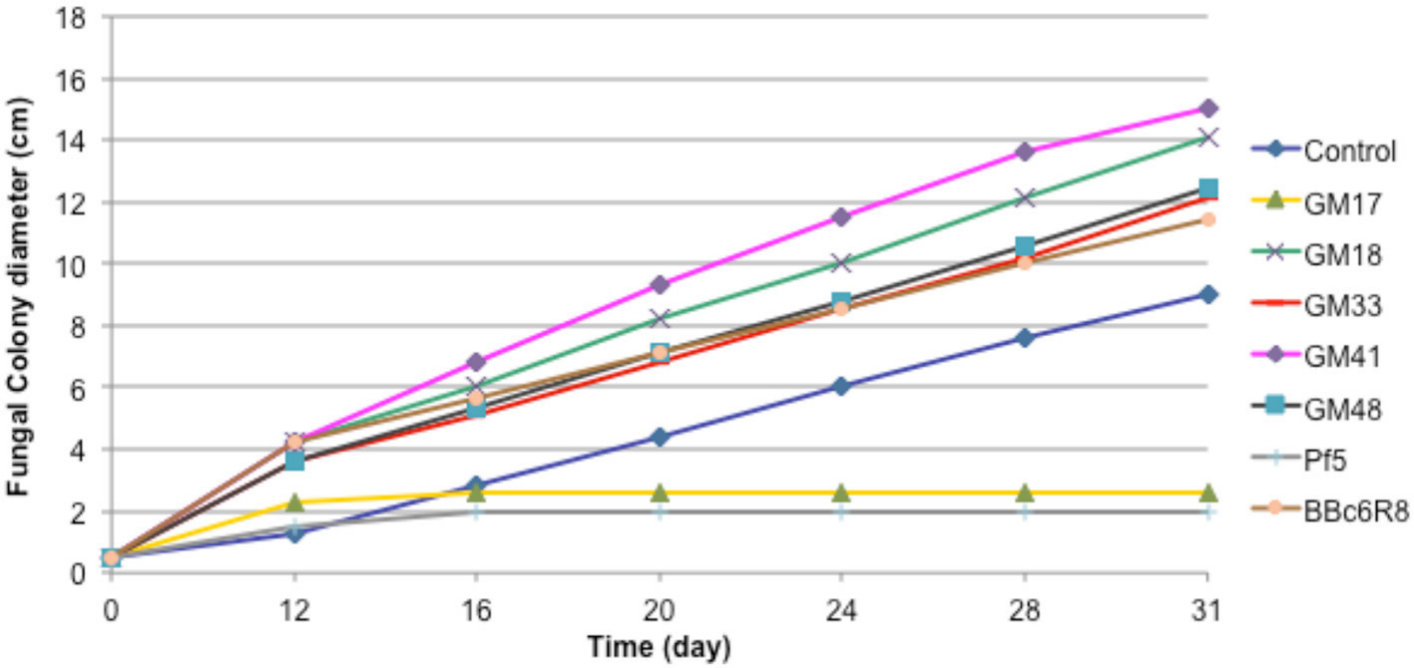
**Effect of seven *Pseudomonas* strains on radial growth of *Laccaria bicolor* S238N**. Control: bacterial suspension was substituted by sterile deionized water. Each data point represents the mean (±SE) of seven replicates.

We carried out *in vitro* co-cultures of 23 *Pseudomonas* isolates and *L. bicolor* on which we measured the mycelium radial growth, apex number, branching angle and branching density. The radial growth of *L. bicolor* S238N was differentially influenced by these 23 *Pseudomonas* strains, including the two controls. Based on the wide array of growth effects on *L. bicolor* S238N, *Pseudomonas* strains were categorized into three classes—(1) no significant effect, (2) a significant negative effect, and (3) a significant positive effect (Figure 1, Table 1, Table S1). Indeed, compared to the control, strains GM16 and GM24 had no significant effects on fungal radial growth (Table S1). Alternatively, strains Pf-5 and GM17 significantly inhibited *Laccaria* radial growth as early as 12 d of dual cultivation (Figure 1, Table 1, Table S1) at which point no contact between *L. bicolor* mycelium and bacterial colonies was observed. In contrast, the MHB strain BBc6R8 stimulates fungal growth at 12 d and 14 d, as well as after contact (Figure 1, Table 1, Table S1). Strains GM18, GM21, GM25, GM30, GM33, GM41, GM48, GM49, GM50, GM55, GM60, GM67, GM74, GM78, GM79, GM80, GM84, and GM102 each stimulated *L*. *bicolor* growth after 16 d (Table 1, Table S1). Strains GM41 and GM18 had the most significant effect on *L. bicolor* growth, habit and colony diameter throughout the experiment (Figure 1 and Table S1). Most of the above strains had a similar positive effect on apex number, branching angle and branching density (Table 1), though GM41 had a slightly higher enhancement of apex number. However, both GM41 and GM18 also increased hyphal branching angle and branching density. By contrast GM17 and Pf5 induced a high amount of curvature in the hyphae. Interestingly, hyphal branching density was higher with GM17 than with BBc6R8; hyphal branching density was considerably reduced in the presence of Pf-5.

**Table 1:** **Summary of the *Pseudomonas* strains effect on *Laccaria bicolor* S238N-mycelial growth, *L. bicolor* gene expression, and mycorrhization of *Populus* roots**.

**Bacterial treatment**	**Fungal colony diameter**	***L. bicolor* gene expression**	***Mycorrhization***
GM16	Neutral		
GM17	−	*Tra1(−) Tect2(−) Gcn5(−) Cipc1(−)*	−
GM18	+	*Tra1(+) Tect2(+) Gcn5(+) Cipc1(+)*	+
GM21	+		
GM24	Neutral		
GM25	+		
GM30	+	*Tra(+) Tect2(+) Gcn5(+) Cipc1(+)*	−
GM33	+		
GM41	+	*Tra(+) Tect2(+) Gcn5(+) Cipc1(+)*	+
GM48	+		
GM49	+		
GM50	+		
GM55	+		
GM60	+		
GM67	+		
GM74	+		
GM78	+		
GM79	+		
GM80	+		
GM84	+		
GM102	+		
Pf-5	−	*Tra1(−) Tect2(−) Gcn5(−) Cipc1(−)*	−
BBc6R8	+	*Tra1(+) Tect2(+) Gcn5(+) Cipc1(+)*	+

*(+), up-regulated; (−), down-regulated*.

### L. bicolor gene expression analysis

We evaluated the expression of S238N genes (Figures S1, S2), *Cipc1, Tectonin2, Fox2, Tra1, Gcn5, Spt3*, and A*da3*, which are known marker genes for *P. fluorescens* BBc6R8 interactions with *L. bicolor* (Deveau et al., 2007) at 14 DPI before *Laccaria*-bacteria contact and at 21 DPI after extended contact. A wide variation in the expression of the seven *Laccaria* target genes was observed during co-cultivation experiments, before, as well as after, contact with the bacterial strains. However, strains GM17, GM33, GM41, GM48, Pf-5, and BBc6R8 consistently and significantly modified (i.e., some up-regulated and some down) the expression of *Tra1, Tectonin2, Gcn5*, and *Cipc1* both before and after contact. We did not detect any significant change in expression in *Ada3, Fox2*, and *Spt3* across all bacterial strains at either time point (Figures S1, S2).

### Bacterial effect on *populus* root architecture

**Figure 2:**
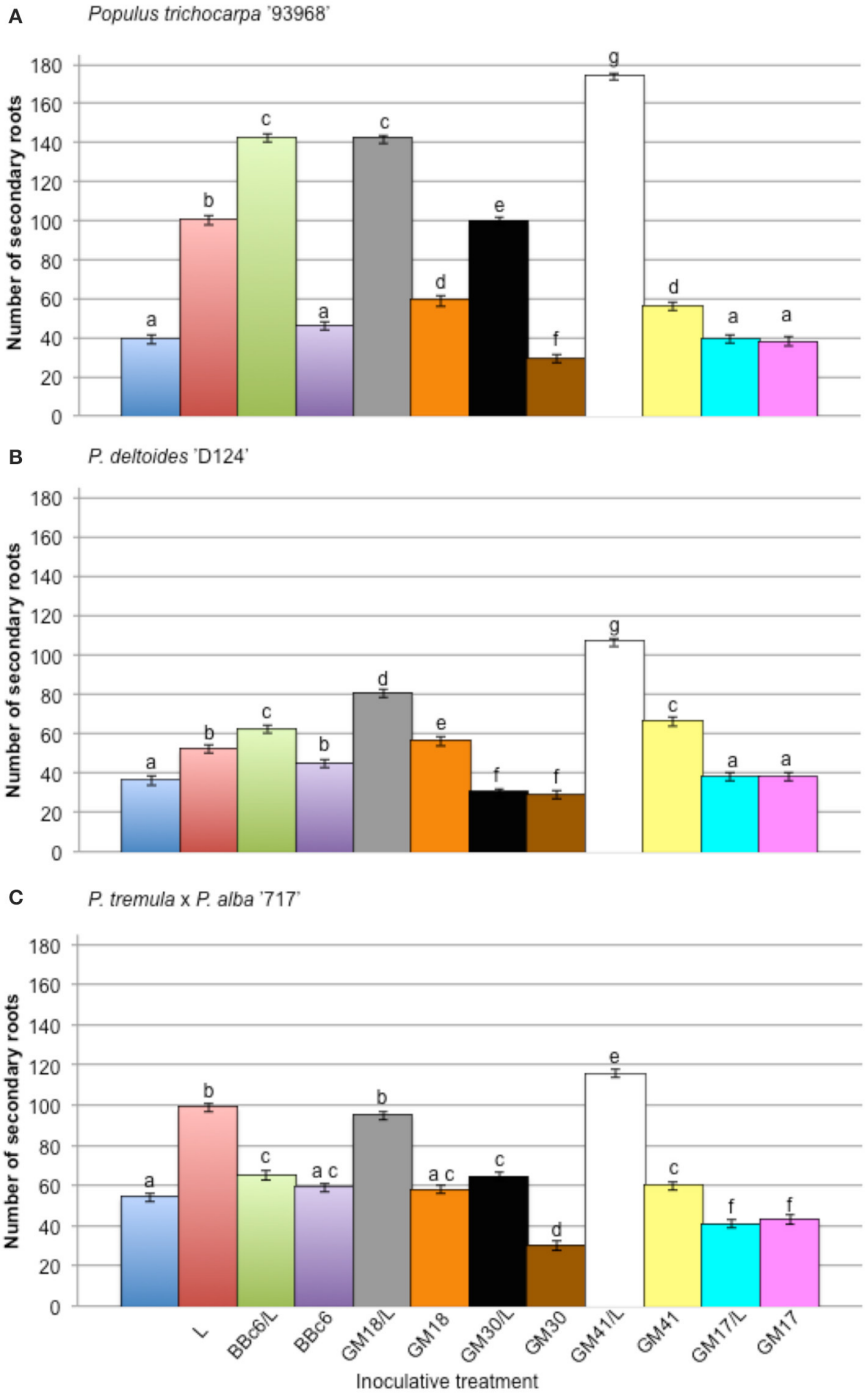
**Effect of *Pseudomonas fluorescens* strains BBc6R8, GM18, GM30, GM41, and GM17 on the number of secondary roots of *Populus*, 31 days post-inoculation (DPI)**. **(A)** *Populus trichocarpa* ‘93-968,’ **(B)** *P. deltoides* ‘D-124’ and **(C)** *P. tremula* x *P. alba* ‘717-1B4’. “L”: *Laccaria bicolor* S238N. Bars with the same letters are not significantly different according to Tukey's HSD test. Error bars denote standard error.

The effect of bacterial strains BBc6R8, GM41, GM30, GM18, and GM17 in the presence of *L. bicolor* S238N on the number and length of secondary roots across three *Populus* genotypes, “93-968,” “717-1B4,” and “D-124,” was evaluated *in vitro* at 14 DPI and 31 DPI (Figures S2, S3). In the presence of *Laccaria* alone, and for plants growing with *Laccaria*-GM18, *Laccaria*-GM30, and *Laccaria*-GM41, the number of secondary roots is significantly higher than for plants growing axenically at 14 DPI and 31 DPI (Figures S2, S3). However, the three *Populus* genotypes in these three tripartite co-conditions have more secondary roots than plants treated by *Laccaria* alone. In fact, plants treated by *Laccaria*-GM41 produced more secondary roots than any other fungal-bacterial combinations (Figure 2). “D-124” plants have longer secondary roots when treated by *Laccaria*-GM30 than “93-968” and “717-1B4” (Figures S4, S5). ANOVA result showed consistent *Populus* genotype effects (Table 2).

**Table 2:** **ANOVA results for the percentage of mycorrhizal colonization (%Myc) in three *Populus* genotypes: *Populus deltoides* “D-124,” *P. trichocarpa* “93-968,” and *P. tremula* x *P. alba* “717-1B4”**.

**Trait**	**Effect**	***df***	***F*-value^*a*^**	***P***
% Myc	Genotype	2	5.26	0.0001
	Bacterial treatment	4	3.38	0.2354
	Block	7	0.2975	0.3456

a *Pooled error was used as the denominator in all F-tests*.

### Bacterial effect on *L. bicolor* colonization of *populus* roots

**Figure 3:**
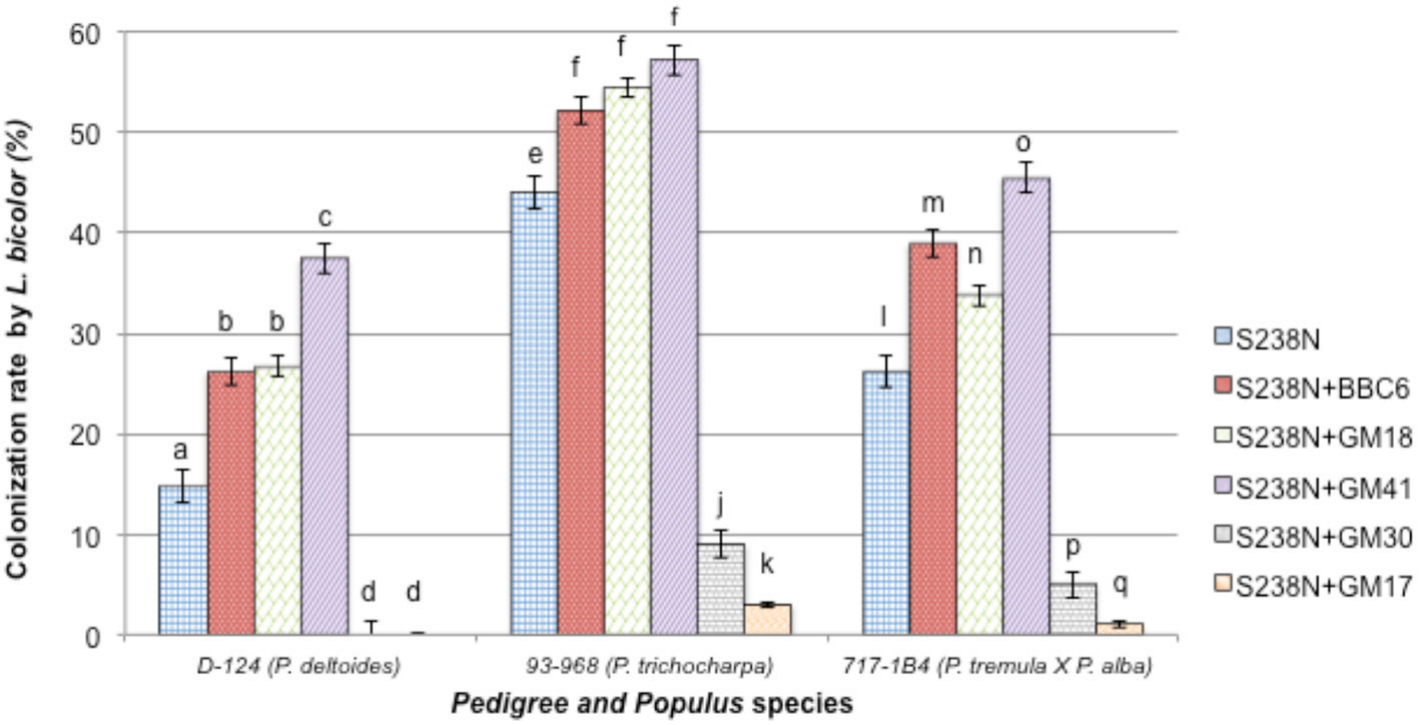
**Percentage of roots from three *Populus* genotypes colonized by *Laccaria bicolor* S238N in co-culture with *Pseudomonas fluorescens* strains BBc6R8, GM18, GM41, GM30, and GM17**. Bars with the same letters are not significantly different according to Tukey's HSD test. Error bars denote standard error.

Twelve weeks after inoculation, *L. bicolor* colonization of the plant root system was assessed. No colonization by potential contaminant was noticed either by microscopic observation or re-isolation experiments. The root systems of “93-968” clones (*P. trichocarpa*) were colonized with an average rate of 44 ± 1%, while “D-124” (*P. deltoides*), and “717-1B4” (*P*. *tremula* × *alba*) plants were colonized at 15 ± 2% and 26 ± 1%, respectively (Table 2, Figure 3). Under bacterial treatment, “93-968” roots were the most efficiently colonized, followed by “717-1B4” and then “D-124.” Across the three *Populus* genotypes, the highest *Laccaria* colonization rate occurred in combination with GM41. In contrast, GM30 and GM17 produced some of the lowest colonization rates, i.e., 9 ± 2% (with GM30) and 3 ± 2% (with GM17) with “93-968,” and 5 ± 2% (with GM30) and 1 ± 2% (with GM17) with “717-1B4,” while “D-124” was not colonized by *Laccaria* in combination with either GM17 or GM30.

## Discussion

Here we investigated the effect of 21 *Pseudomonas* strains, isolated from native *P. deltoides* rhizosphere, on *L. bicolor* growth, morphology and *Populus* root colonization in order to characterize MHB strain within the model system *Populus*-*Laccaria*. Our results demonstrate potential use of microbial component for improved root biomass production. The inferred data suggest that particular microbial combinations might determine which interactions can be exploited to enable inoculated plants to behave in a more competitive way and to survive when established in the field. This idea is supported by the work of Requena et al. (1997), in which *Rhizobium* strains from the rhizosphere of the legume *Anthyllis cystoides* was screened and combined with arbuscular mycorrhizal fungi. They highlighted that beneficial fungi where more effective in promoting plant growth when associated with rhizobacteria from the pool of native soil bacteria, which supposedly are adapted to the particular soil and climatic conditions of an area.

Deveau et al. (2007) previously demonstrated that *P. fluorescens* BBc6R8 has a stimulating effect on *L. bicolor* S238N growth, hyphal branching angle and density and gene expression in an agar plate system. Here, we confirmed that strain BBc6R8 has such a helper effect on *L. bicolor* S238N. Additionally, we determined that 17 out of 21 *Pseudomonas* isolates (GM21, GM25, GM30, GM48, GM49, GM50, GM55, GM60, GM67, GM74, GM78, GM79, GM80, GM84 GM102, GM18, and GM41) have a positive effect on the growth of the same fungal strain. Fifteen of the 21 bacterial isolates have a similar effect as BBc6R8 and two, GM18 and GM41, have a more pronounced effect on fungal growth. Similar to BBc6R8, we demonstrated that GM18 and GM41 increase colony growth by increasing hyphal density via hyphal branching. Enhanced hyphal branching represents a potential mechanism that could lead to increased colonization of plant roots by ectomycorrhizal fungi. Aspray et al. (2013) have suggested that *P. fluorescens* strains might be able to produce branching factor(s) as reported in the MHB *Paenibacillus* spp. EJP73. Unexpectedly, we observed a simultaneous increase in mycelium growth and density, suggesting that the mechanism and molecular regulators may differ. We also report a negative effect on *L. bicolor* S238N growth in combination with strain GM17 as well as for the biocontrol strain Pf-5. We observed the production of phenazine crystals in GM17 culture (Ovchinnikova et al., 2014), which has been shown to provide biocontrol through antimicrobial activity (Shanmugaiah et al., 2010). Interestingly, GM17 produced reduced-radial colony growth and increased branching density of *L. bicolor* similar to that reported by Aspray et al. (2013) for *Paenibacillus* sp. EJP73 and *Lactarius rufus*. Schrey et al. (2007) also demonstrated that the mycorrhiza helper bacterium *Streptomyces* spp. AcH 505 with the ectomycorrhizal fungus *Amanita muscaria* reduces the hyphal diameter. Compounds released by the bacterium into the growth medium were sufficient to induce these changes. Furthermore, the promoted growth rate and reduced diameter of hyphae are associated with a high frequency of hyphal tips with reorganized actin cytoskeleton. Schrey et al. (2007) suggest that bacterial compounds modulate the actin organization in the hyphal tip region, which could in turn induce the morphological changes. Hence, the resulting enhanced hyphal growth might be an important factor behind the mycorrhiza helper effect exhibited by some MHB.

We confirmed the responsiveness of the *L. bicolor* S238N genes *Cipc1, Tectonin2, Fox2, Tra1, Gcn5, Spt3*, and A*da3* upon co-cultivation with tested *Pseudomonas* isolates, including the known MHB strain BBc6R8. Despite wide variation in gene expression levels across the bacterial strains, we were able to demonstrate significant changes (< or >2-fold) in expression for *Tra1, Tectonin2, Gcn5*, and *Cipc1*, before and after contact with GM17, GM33, GM41, GM48, Pf-5, and BBc6R8. Given that these gene expression patterns appear to be shared across the *Pseudomonas* strains, and not specific to the *L. bicolor* S238N-BBc6R8 interaction, we propose that these genes could be considered as marker/reporter genes of *L. bicolor* S238N-*P. fluorescens* interactions.

The predicted function of each of these genes varies. For example, *Tra1* is known to be involved in transcriptional regulatory complexes (SAGA, SALSA) in yeast (Wu et al., 2004), suggesting that chromatin structure modifications take place in *L. bicolor* S238N in response to *Pseudomonas*. A similar mechanism can be proposed for *Gcn5* (the SAGA complex: Spt-Ada-Gcn5–acetyltransferase). *Tectonin2* codes for a protein known to participate in bacterial cell aggregation during phagocytosis by amoebae cells (Huh et al., 1998) and could play a role in cell recognition and/or fungal cell interaction with *Pseudomonas*. As reported by Deveau et al. (2007), *Cipc1* appears to be linked to the modification of *L. bicolor* growth and morphology and could therefore be considered a marker of induction of the pre-symbiotic status of the fungus (opposed to the alternate saprobe status of the exploring mycelium). In support of these putative roles, we observed that with the biocontrol strains GM17 and Pf-5 there was a down-regulation of all four genes, *Tra1, Tectonin2, Gcn5*, and *Cipc1*, and an associated negative effect on fungal growth. Finally, we noticed the strongest effect on fungal radial growth from strains GM41 and GM18. These two bacterial isolates had a wide variation on the expression level of *Tra1, Tectonin2*, and *Gcn5*, suggesting that gene induction may not be quantitatively linked to the highest phenotypic effect and that there may be a potential role for post-transcriptional regulation during colonization and signal transduction. Further investigation will be needed to confirm this gene expression to phenotype relationship.

We next examined the effect of BBc6R8, GM17, GM18, GM30, and GM41 on *in vitro* root architecture (number and length of secondary roots) across three *Populus* genotypes representing alternate *Populus* species. We focused on four *Pseudomonas* isolates—GM17 because of its negative effect on *Laccaria*, GM30, and the GM18 because of the similar effect as BBc6R8 and GM41 because of its strong positive effect on the *L. bicolor* S238N growth. We have shown that BBc6R8, GM18, GM30, and GM41 significantly enhanced the number of secondary roots across all tested genotypes in the presence of *L. bicolor* S238N. Poole et al. (2001) reported that bacteria (*Burkholderia* and *Pseudomonas*) isolated from *Pinus sylvestris*—*Lactarius rufus* mycorrhizas did not affect the total number of roots formed by the plants but only ectomycorrhizal formation. While Bending et al. (2002) found bacteria isolates (*Burkholderia* spp., *Serratia* spp., *Pseudomonas* spp., and *Bacillus* spp.) from *Pinus sylvestris*—*Suillus luteus* mycorrhizas stimulating root growth and even shoot growth by *Bacillus* spp. but not ectomycorrhiza.

Bending et al. (2002) also demonstrated that *Pseudomonas* isolates stimulated lateral root formation similar to our results. In total, these results suggest a diversity of growth promoting mechanisms operating on plant or fungi and/or both and illustrate the complexity of the evolving MHB concept.

The lack of S238N colonization of *P. deltoides* roots and the low colonization rates of the two other *Populus* genotypes observed in tripartite combinations with strain GM30 in the greenhouse was unexpected, given that in the *in vitro* studies GM30 had a beneficial effect on *L. bicolor* or *Populus* growth in binary combinations. Bending et al. (2002) have reported similar observations and showed that several bacterial isolates enhanced plant growth substantially, although these effects were unrelated to either root colonization by the fungus or ectomycorrhiza formation. Comparative variation in *P. trichocarpa* and *P. deltoides* colonization by *L. bicolor*, with *P. trichocarpa* obtaining higher colonization rates, and as previously observed in several studies (Tagu et al., 2005; Labbé et al., 2011), remained generally consistent across bacterial treatments. That is, strains BBc6R8, GM18, and GM41 enhanced colonization yield in both *P. trichocarpa* and *P. deltoides* in relative abundance, interestingly GM41 increased the colonization rate of *P. deltoides* by 2.5 fold.

Labbé et al. (2011) has suggested that the activation of defense mechanisms in *P. deltoides* may inhibit *L. bicolor* colonization, while in *P. trichocarpa* defense mechanisms are ultimately repressed. Similarly, Garbaye (1994) suggested that a mycorrhiza helper bacterium could increase the rate of plant root colonization by positively affecting “root receptivity.” Lehr et al. (2006) reported such an observation with AcH505, which suppresses defense response in Norway spruce (*Picea abies*) allowing colonization by *Heterobasidion annosum*. We propose that the tested *Pseudomonas* strains might play a similar role with a variety of responses.

With respect to bacterial strain origins, BBc6R8 was isolated from a sporocarp of *L. bicolor* S238N associated with Douglas-fir roots (Duponnois and Garbaye, 1991) while strains GM18 and GM41 were isolated from roots of native *P. deltoides* (Gottel et al., 2011). Each of these strains improved the *L. bicolor* S238N colonization of *P. deltoides*. However, strain GM41 produced significantly higher *L. bicolor* colonization of *P. deltoides* and might potentially be recruited by *P. deltoides* and may have co-evolved within *Laccaria bicolor* and *Populus deltoides* system. Duponnois et al. (1993) have suggested the high fungal specificity of MHB is controlled by the bacterial and fungal relationship and not the tree species. Our work suggests that the *Laccaria*-MHB interactions are not as specific as believed, at least at the isolate level. This is in accordance with Founoune et al. (2002a) work in which bacterial inoculant has been used in Australian *Acacia* species and with their mycorrhizal associates.

To date, most MHB reports have investigated the behavior of fungal mycelium correlated with efficiency of mycorrhiza formation (Garbaye and Duponnois, 1993; Frey-Klett et al., 1997; Poole et al., 2001; Founoune et al., 2002a). As suggested by Garbaye (1994), the mycorrhizal helper effect could include increased receptivity of roots to fungal hyphae or improved fungal colonization of fine roots due to plant cell wall modifying bacterial substances. In general our MHB strains that stimulated mycelial growth and mycorrhiza formation also acted as plant growth promoters (i.e., increased root branching) suggesting auxin-like activity. Production of auxin or indole-acetic acid (IAA) is known to be widespread among rhizosphere bacteria and is one of the major marker genes by which the beneficial effect of plant-promoting bacteria is measured (Glick, 1995). Auxin production has been previously reported in BBc6R8 (Frey-Klett et al., 2007) and we have detected auxin production in our *Pseudomonas* strains (data not shown). *L. bicolor* S238N also stimulates lateral *Populus* root production via fungal auxin and volatile molecules (Felten et al., 2010). It is reasonable to speculate that enhanced lateral root formation in response to beneficial microbes may be a conserved mechanism that soil microbes employ for their own benefit in order to enhance root exudation and thus increase the energy flow from the roots of host plants.

Although it has been suggested that the predominant mechanism to describe the mycorrhizal helper effect is the positive effect on fungal metabolism and morphology, MHB could also promote plant growth, in contrast to Duponnois et al. (1993). In the case of stimulation of *L. bicolor* mycorrhiza formation on Douglas-fir by BBc6R8, fungus growth was found to be inhibited as the bacterial inoculum increased, so that low doses were the most stimulatory to the fungus (Frey-klett et al., 1999). As Bending et al. (2002) have suggested, characterizing the spatial distribution and size of bacterial communities within the rhizosphere will be required to determine the nature and extent of bacterial effects on mycorrhiza formation and plant growth.

In conclusion, we report that MHB strains isolated from native *Populus* rhizosphere samples have strong benefits on *L. bicolor* growth and root colonization of *Populus* trees. This study reflects results mainly obtained from *in vitro* and greenhouse experiments. Further investigations are needed to explore whether these systems behave similarly in nursery and/or field studies. This work emphasizes the importance of studying MHB since plant-fungal interactions can be strongly influenced by MHB and may therefore be key players in determining rhizosphere diversity and thus a molecular level of understanding is necessary engineering a sustainable environment.

### Conflict of interest statement

The authors declare that the research was conducted in the absence of any commercial or financial relationships that could be construed as a potential conflict of interest.
