# Supplementary material for: Newly identified helper bacteria stimulate ectomycorrhizal formation in Populus
Source: Front Plant Sci. 2014 Oct 24;5:579. doi: 10.3389/fpls.2014.00579 (PMC4208408; doi:10.3389/fpls.2014.00579)
Supplement: Supplementary file 1 [file DataSheet1.DOCX]

***Supplementary Material***

**Newly identified helper bacteria stimulate ectomycorrhizal formation in *Populus***

***Jessy L. Labbé^*^, David J. Weston, Nora Dunkirk, Dale A. Pelletier, Gerald A. Tuskan***

*Biosciences Division, Oak Ridge National Laboratory, Oak Ridge, TN, USA.*

*** ***Correspondence****:*

*Jessy Labbé*

*Oak Ridge National Laboratory*

*Biosciences Division*

*Oak Ridge, TN 37831-6407*

*Tel: +1 865 576 3478*

*Fax: +1 865 576 9939*

[*labbejj@ornl.gov*](mailto:labbejj@ornl.gov)

## Suplementary Tables


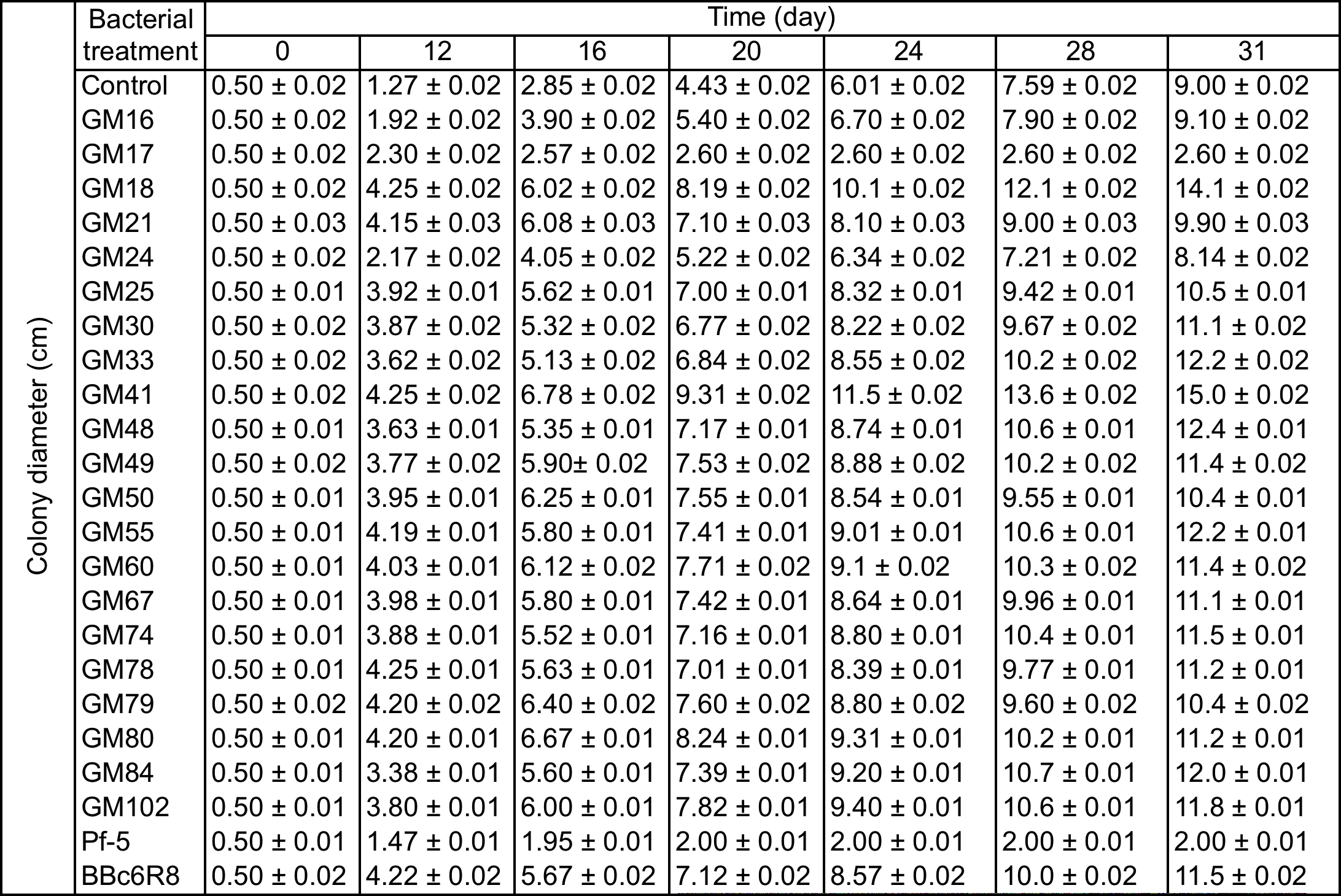


**Supplement Table1. Effect of 23 bacterial strains on radial growth of *Laccaria bicolor* S238N, in pairwise culture.** Control: bacterial suspension was substituted by sterile deionized water. Each point is the mean (±SE) of seven replicates.

## Supplementary Figures

**
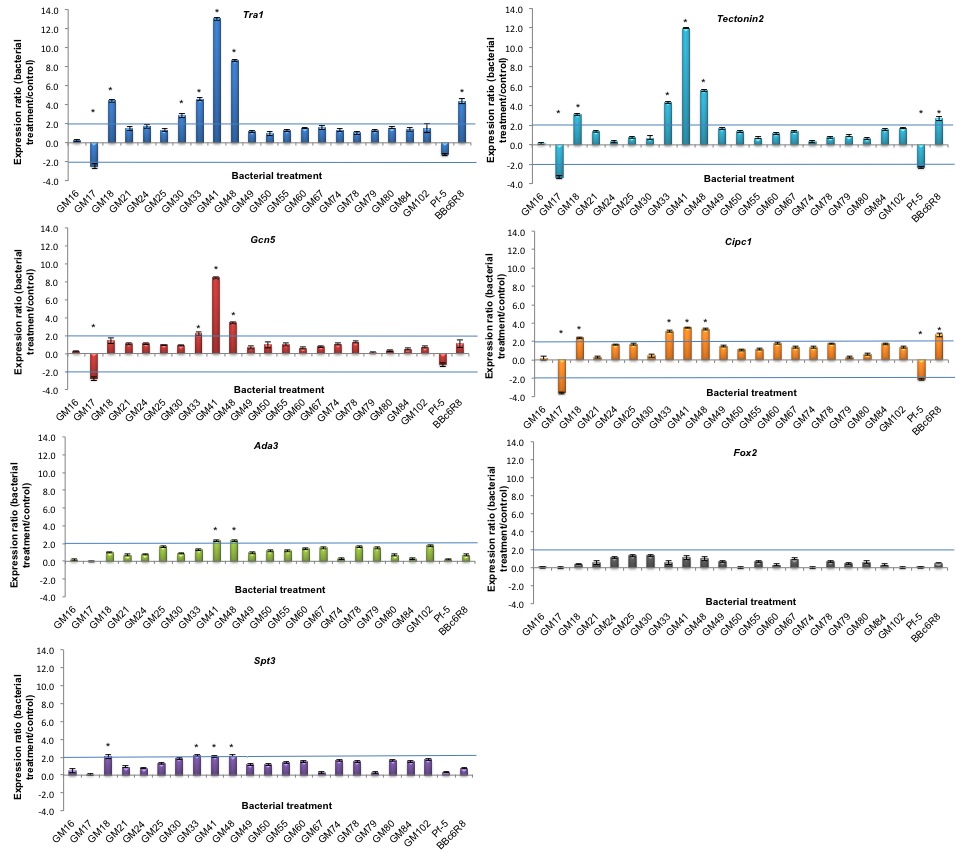
**

**Supplement Figure 1.** **Gene expression analysis of seven *Laccaria bicolor* ‘S238N’ genes responsive to the helper bacteria *Pseudomonas fluorescens* ‘BBc6R8’, 14 (DPI) days after co-culture with 23 different *Pseudomonas* strains (X axis).** * Significant. Bars represent the expression ratio between bacterial treatment and control (±SE).


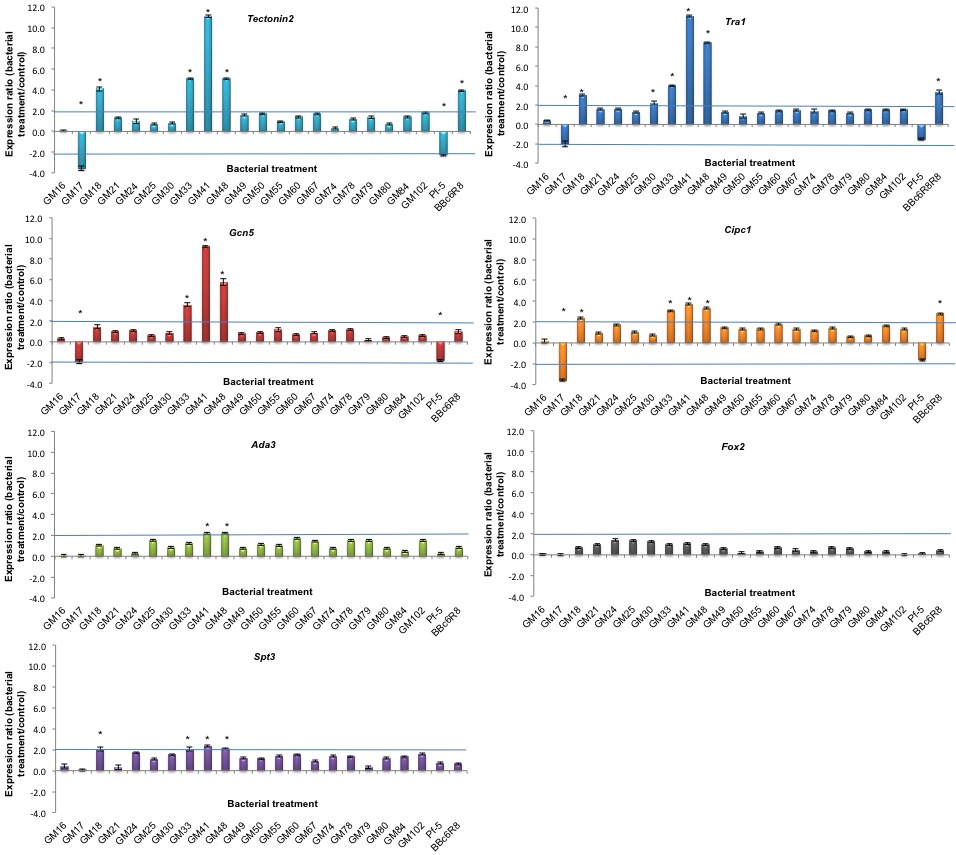


**Supplement Figure 2.** **Gene expression analysis of seven *Laccaria bicolor* ‘S238N’ genes known to be responsive to the helper bacteria *Pseudomonas fluorescens* ‘BBc6R8’, 21 (DPI) days after co-culture with 23 different *P. fluorescens* bacterial strains (X axis).** *Significant. Bars represent the expression ratio between bacterial treatment and control (±SE).


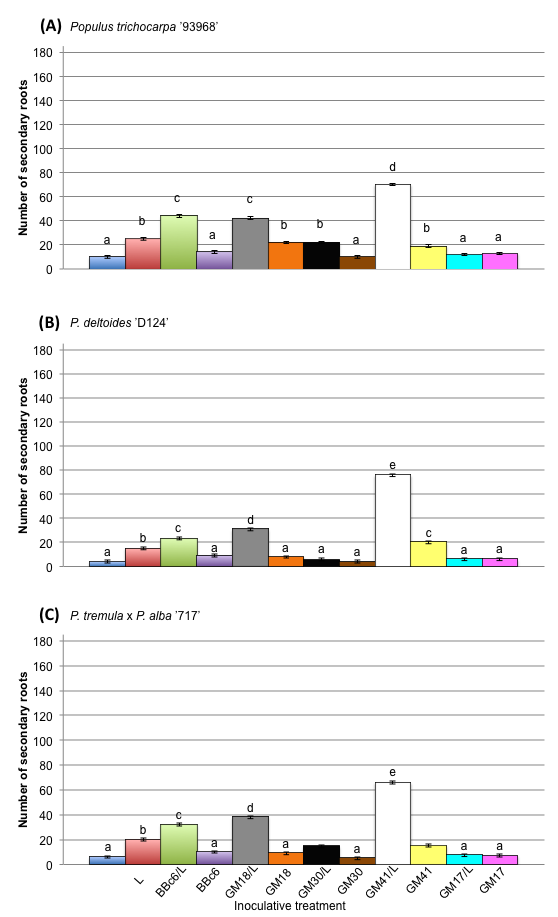


**Supplement** **Figure 3.** **Effect of *Pseudomonas fluorescens* strains BBc6R8, GM18, GM30, GM41 and GM17 on the number of secondary roots of of** ***Populus***, 14 days post inoculation (DPI). “L”: *Laccaria bicolor* S238N. Bars with the same letters are not significantly different according to Tukey’s HSD test. Error bars denote standard error.


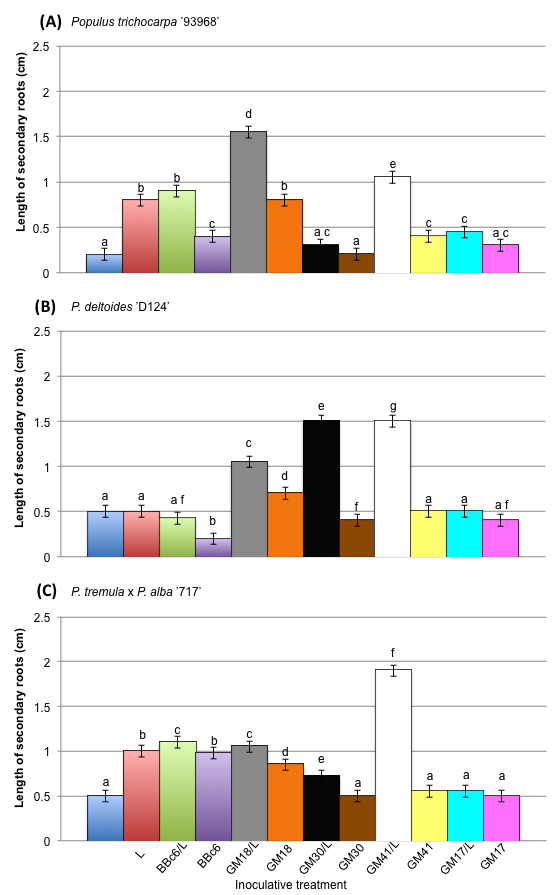


**Supplement Figure 4.** **Effect of the *Pseudomonas fluorescens* strains BBc6R8, GM18, GM30, GM41 and GM17 on the length of secondary roots of of** ***Populus***, 14 days post inoculation (DPI). “L”: *Laccaria bicolor* S238N. Bars with the same letters are not significantly different according to Tukey’s HSD test. Error bars denote standard error.


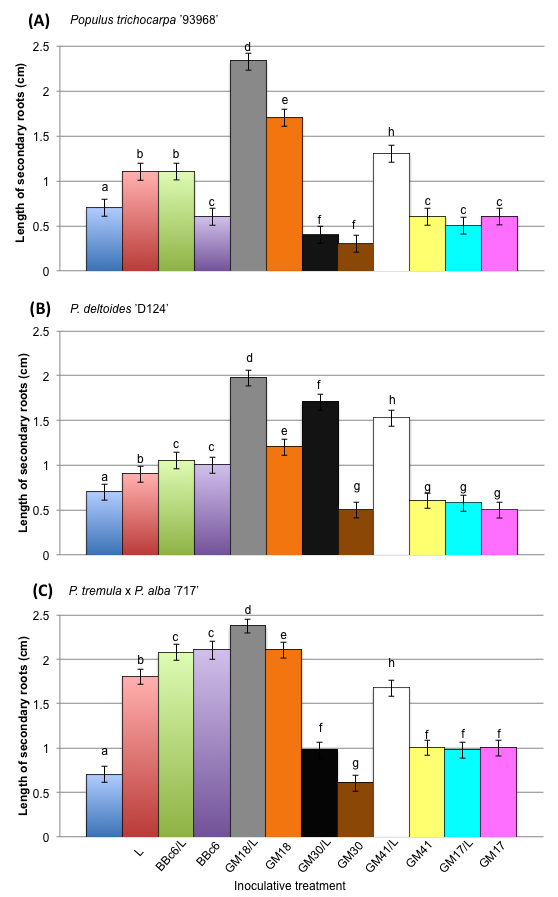


**Supplement Figure 5.** **Effect of the *Pseudomonas fluorescens* strains BBc6R8, GM18, GM30, GM41 and GM17 on the length of secondary roots of of *Populus*,** 31 days post inoculation (DPI). “L”: *Laccaria bicolor* S238N. Bars with the same letters are not significantly different according to Tukey’s HSD test. Error bars denote standard error.
